# Supplementary material for: A Global Screen for Assembly State Changes of the Mitotic Proteome by SEC-SWATH-MS
Source: Cell Syst. 2020 Feb 26;10(2):133–155.e6. doi: 10.1016/j.cels.2020.01.001 (PMC7042714; doi:10.1016/j.cels.2020.01.001)

Q92506 | DHB8_HUMAN | HSD17B8 FABGL HKE6 RING2 SDR30C1
Monomer MW [kDa]: 26.974 Monomer expected elution fraction: 52

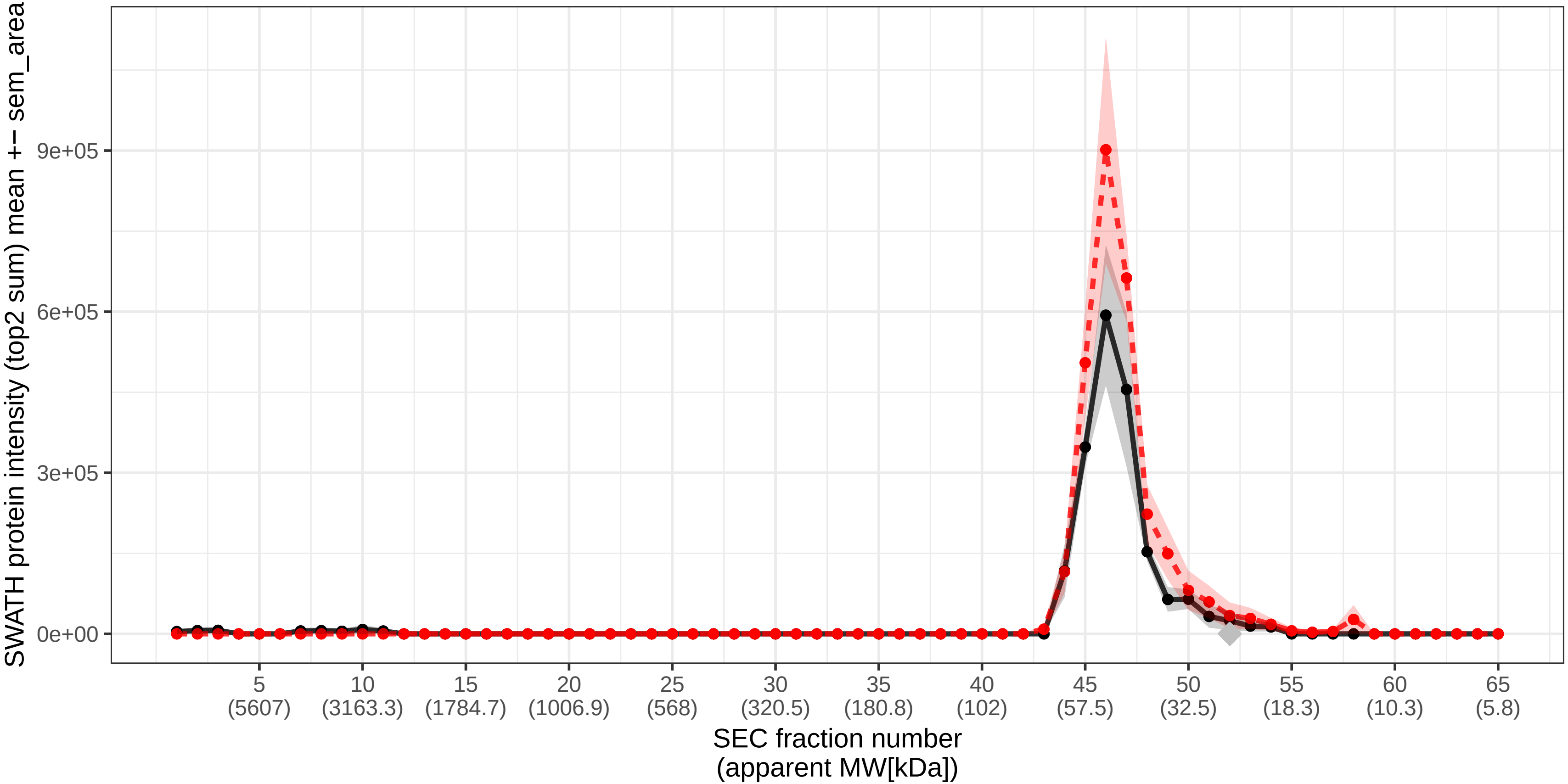

Supplement: Data S1. SEC-SWATH-MS Protein Chromatograms, Related to Figure 1 [file mmc6.zip › SECchrom_Q92506_DHB8_HUMAN_HSD17B8_FABGL_HKE6_RING2_SDR30C1.pdf]
